# Advancing SSP-aligned scenarios of shipping toward 2050

**DOI:** 10.1038/s41598-024-58970-3

**Published:** 2024-04-18

**Authors:** Diogo Kramel, Sebastian M. Franz, Jan Klenner, Helene Muri, Marie Münster, Anders H. Strømman

**Affiliations:** 1 Industrial Ecology Programme (IndEcol), Norwegian University of Science and Technology (NTNU), Trondheim, Norway; 2 Department of Technology, Management and Economics, Technical University of Denmark (DTU), Copenhagen, Denmark

**Keywords:** Gravity modeling, Shipping scenarios, SSP, Energy demand, Spatial inventory, Environmental impact, Climate change

## Abstract

Developing comprehensive scenarios for the shipping sector has been a challenge for the Integrated Assessment Model (IAMs) community, influencing how attainable decarbonization is in the sector, and for Earth System Models (ESMs), impacting the climate contribution of shipping emissions. Here we present an approach to develop spatially explicit energy demand projections for shipping in alignment with the Shared Socioeconomic Pathways framework and IAMs projections of global fossil fuel demand. Our results show that shipping could require between 14 and 20 EJ by 2050, corresponding to a 3% and 44% increase from 2018 for the SSP1-1.9 and SSP3-7.0 scenarios. Furthermore, the energy projections we present in this publication can be combined with different fuel mixes to derive emission inventories for climate modeling and, thus, improve our understanding of the various challenges in mitigating emissions for shipping. Through that, we aim to present a framework to incorporate detailed spatial shipping inventories and increase transparency for the scientific community.

## Introduction

Climate research relies on the combined assessment of environmental and human systems, which can be achieved through integrated assessment models (IAMs) to understand the anthropogenic drivers of climate change and Earth system models (ESMs) to assess the impact on the natural systems. The existing feedbacks between these two modeling approaches in climate science require a spatial component to capture local and global climate responses. This can be especially difficult for highly mobile sectors like shipping that span across all regions, including climate-sensitive regions such as the Arctic. Moreover, the maritime sector has been considered one of the hardest sectors to abate emissions in transportation^1–4^ and likely a late decarbonizer if we are to stabilize global warming to 1.5 or 2 degrees, despite the recent progress made at COP28 and the revised targets by IMO to reach net-zero GHG emissions from international shipping around 2050. By 2023, it is still dominated by fossil fuels, partly because direct electrification has limited applications and carbon-free fuels are not yet technologically mature or globally available for bunkering. Around 70% of the fleet today operates with heavy fuel oils (HFO) that consist of a byproduct of oil refineries with low added value, meaning that any alternative fuel would increase operation costs in the sector. Thus, the current one billion tonnes of CO_2_ emitted annually by shipping, which corresponds to around 2.5% of the world’s emissions^5^, could increase by 85% by 2050 in a business-as-usual scenario^6^, making shipping an increasingly critical sector as other sectors succeed in reducing their emissions in the coming years.

Scenarios are vital to understanding the future of shipping, a sector closely linked to changes in the trade dynamics that can change substantially in the period of multiple decades and centuries in which climate projections are carried out. Some key drivers of change in the past years that might persist include the gradual relocation of supply chains towards Eastern Asia^7^, e.g., growth of China’s share of international trade from 2.3 to 11.3% between 1992 and 2012^8^; increase in shipping in the Arctic^9,10^; the transition towards decentralized sources of green fuels instead of centralized extraction of fossil fuels^4^. If efforts are pursued to phase out fossil fuel, oil tankers are likely to experience a decrease in demand, whereas novel ship types designed to carry the fuel demand of the future are to be deployed, as around 44% of fossil fuels are currently transported by sea^11^.

To understand how those changes can affect shipping, several studies have investigated the linkage between socioeconomic factors and international trade, traditionally using linear regressions^12^ or the theory of gravity modeling^5,13,14^. In these studies, the bilateral trade of each country-pair is found to be proportional to the product of their gross domestic product (GDP), to commonly shared traits such as language, past colonizers, and regional trade agreements (RTA), and indirectly proportional to the cost of transportation often as a function of distance, even though freight costs have decreased significantly reducing the barrier effect of this indicator^15^. Thus, trade being highly dependent on GDP and population, recent studies have attempted to link the changes in shipping traffic to the projections of main socioeconomic drivers as illustrated by the shared socioeconomic pathways (SSPs)^16^. The SSP framework provides an extensive description of qualitative and quantitative indicators aligned with different forecasts for the global development^16^, even though most studies opt for using single elements from these narratives, such as GDP and population^17^. They are also a fundamental part of scenario analysis carried out by IAMs that combine different sectors in society (e.g., industry, transport, agriculture) and their climate impact (e.g., pollution, emissions) for understanding the impact of society on the climate^18–20^.

The climate change implications stemming from these sectors can be thoroughly investigated through the use of climate models (e.g., regional climate models, Earth system models) based on the increase in anthropogenic emissions and concentration of GHG gases and aerosols in the atmosphere. The time and the local in which those emissions occur are essential to carry out accurate simulations^21^, which require detailed emission inventories of anthropogenic emissions, including from shipping. The use of bottom-up emission inventories in many disciplines led to several attempts to develop scenarios specifically for shipping. Early attempts include investigating the increase in ship demand based on a linear relationship between global GDP and international trade^12^, which was later found to be non-linear^22^, for 16 different scenarios that combined the Special Report on Emissions Scenarios (SRES) by the IPCC with different technological pathways, providing energy demand estimation between 16.5 and 29.7 EJ by 2050 (402–725 Mt fuel-Diesel). A subsequent study^9^ have built upon this work and reached similar results of 12.3 and 21.9 EJ (287–510 Mt fuel) for two scenarios through the SeaKLIM model, creating the first bottom-up scenario for shipping. In the 4th IMO GHG study, two different approaches are taken^5^ using i) gravity modeling based on GDP and population projections from the five SSPs, and ii) a logistic regression based on historical trade. Results indicate an energy demand between 15.1 and 20.3 EJ (353–474 Mt fuel) by 2050. More recently, maritime forecast projections^23^ have estimated a much more conservative energy demand peaking at 11.3 EJ towards 2050. In summary, scenarios are found to vary between 11.3 and 29.7 EJ in the literature. However, scenarios and forecasts in the past have considered that globalization and trade will continue to increase at constant elasticities, reaching sometimes four-folding figures by 2050^24^, leading to high energy demands, when in reality, the ratio trade-income elasticity has been in decline since the late 90 s from 2.7 to 1.6^25,26^.

Another question to be addressed is the development of spatially explicit emission pathways for shipping. Attempts to link projections between regions with satellite data (AIS – automated information system) have been carried^27^ out to build a regional inventory in northern Europe, but not linking them to climate narratives. The combination of high spatial–temporal inventories with projections of trade between countries at a global scale has not been attempted, yet it is a concept that was proposed in the past^12^, so that future changes in vessel traffic densities could be captured from ship movement data. This is a significant gap that we aim to fill with this study to ultimately contribute to climate modeling literature by endogenizing the SSP framework in the sector’s demand projections. Thus, linking directly socioeconomic changes to increase in energy demand taking into consideration the efficiency of different ship types and routes between ports. The limited number of studies that carried out energy demand projections for shipping, together with the wide span in results for equivalent scenarios, indicates that more studies could help build more robust projections aligned with climate scenarios already established in the literature, directly affecting our understanding of the climate mitigation challenges in shipping. Thus, this study aims to consolidate shipping scenarios aligned with the SSP scenarios to improve the representation of shipping’s climate impact across time and space and promote transparency for future studies.

## Methods

In this work, we aim to develop projections that cover a range of different possible scenarios in terms of efforts for mitigation and adaptation of climate change. For that, we use the Shared Socioeconomic Pathways framework that encompass population and GDP projections for individual countries. We also include the different demands for coal, oil and gas from IAMs scenarios aligned with different RCP (Representative Concentration Pathways) consistent with greenhouse gas concentration trajectories resulting in a radiative forcing ranging from 1.9 to 8.6 W m-^2^ . Across the eight SSP-RCP marker scenarios, we opt for two scenarios (SSP1-1.9 and SSP3-7.0) with diverging pathways in terms of mitigation efforts. In an SSP1-1.9 scenario, as the world moves in a more sustainable direction with a gradual reduction of fossil fuel production, the economic growth shifts toward a focus on global welfare and a commitment to achieving sustainable development goals. Meanwhile, in an SSP3-7.0 scenario, regional conflicts push countries to focus on domestic and regional issues, favoring energy and food security goals over broader development leading to a decline of technological development in a world with slow economic development and high population increase in developing countries, while the production of fossil fuels increase compared to 2015. The choice of these two narratives also reflects the contrast between ambitious climate policy measures in place for the shipping sector, closer to a SSP1-1.9 pathway, and increasing regional rivalry in geopolitics, not so far from a SSP3-7.0 narrative.

### Trade projections

The gravity model equation can be used to model bilateral trade flows between a country-pair—also called origin and destination (O&D)—and is widely used to investigate trade flows under changing circumstances based on the economic size and the economic barriers between two regions. It was initially formulated by Tinbergen^28^, inspired by Newton’s law of universal gravitation, as shown in Eq. (1).

1 $${{\text{F}}}_{{\text{ij}}}={\text{G}}\frac{{{\text{M}}}_{{\text{i}}}^{{\upbeta }_{1}}{{\text{M}}}_{{\text{j}}}^{{\upbeta }_{2}}}{{{\text{D}}}_{{\text{ij}}}^{{\upbeta }_{3}}}$$

where Fij is the export flow from country i to country j, M represents the economic size of the exporting and importing regions, distance is represented by D, G is a constant, and βk are the model parameters. The logarithm transformation of the gravity model previously presented will result in the following linear equation.

2 $${{\text{lnF}}}_{{\text{ij}}}={\text{G}}+{\upbeta }_{1}{{\text{lnM}}}_{{\text{j}}}+{\upbeta }_{2}{{\text{lnM}}}_{{\text{j}}}+{\upbeta }_{3}{{\text{lnD}}}_{{\text{ij}}}+\varepsilon$$

Over the years, many additions to gravity modeling have been made to improve its robustness, such as the inclusion of multi-lateral resistance and fixed effects^29^ and the border effects and home bias for national and international trade^30^. To prevent results from being potentially impacted by omitted variables^29^, we explore the inclusion of more independent variables (Eq. 3) going beyond the classical gravity model formulation shown in Eq. (2)

3 $${{\text{lnF}}}_{{\text{ij}}}={\upbeta }_{0}+{\upbeta }_{1}{{\text{lnGDP}}}_{{\text{j}}}+{\upbeta }_{2}{{\text{lnGDP}}}_{{\text{j}}}+{\upbeta }_{3}{{\text{lnPOP}}}_{{\text{i}}}+{\upbeta }_{4}{{\text{lnPOP}}}_{{\text{j}}}+{\upbeta }_{5}{{\text{lndist}}}_{{\text{ij}}}+{\upbeta }_{6}{{\text{CL}}}_{{\text{ij}}}+{\upbeta }_{7}{{\text{CB}}}_{{\text{ij}}}+{\upbeta }_{8}{{\text{CCH}}}_{{\text{ij}}}+ {\upbeta }_{9}{{\text{RTA}}}_{{\text{ij}}}+\upvarepsilon$$

The final version of the model includes GDP and POP (population) to measure the economic size, dist is the distance over water weighted by population size in the main cities of the country, and CL, CB, CCH, RTA are binary values (0 or 1) representing a shared common official language, common border, common colonial history and existence of a regional trade agreement, respectively. The residual term ε reflects unmeasured factors. The βk parameters are obtained through an Ordinary Least Squares regression (OLS) based on historical trade data from the CEPII’s dataset^31^. The dependent variable, Fij, corresponds to BACI bilateral trade flows from 1995 to 2019. With this approach, we assume the impact on shipping will be directly proportional to changes in global trade as shipping transports between 85 and 95% of international trade by weight. Goods and commodities have been distinguished in groups allowing the modeling of projections designed explicitly for six ship segments: bulk carriers, chemical tankers,^5^ container ships, liquefied gas carriers, oil tankers, and ro-ro (roll-on/roll-off) ships. This division follows the proposed methodology based on the Harmonized System Codes (HS Code) HS values^5,32^. Thus, the gravity model is run considering the heterogeneity of different cargoes and different ship types. Results of gravity model coefficients (${\beta }_{i}$) and their respective standard deviation in brackets are shown in Table 1. Non-significant coefficients in the regression (< 95%) are excluded from the analysis.

**Table 1:** Coefficient (${\beta }_{i}$) values and their respective p-values in brackets for the gravity model in Eq. 3 obtained through an OLS regression using the CEPII’s dataset.

	Unit and format	Bulk carriers		Chemical tankers		Container ships	
GDP_i_	US$ (thousands) - log	0.81	(0.00)	1.16	(0.00)	1.01	(0.00)
GDP_j_	US$ (thousands) - log	0.83	(0.00)	0.99	(0.00)	1.27	(0.00)
Population_i_	log	− 0.39	(0.00)	− 0.41 (NS)	(0.26)	− 0.05	(0.00)
Population_j_	log	− 0.03	(0.00)	− 0.14	(0.00)	− 0.43	(0.00)
Distance	km – log	− 0.13	(0.00)	− 1.05	(0.00)	− 0.85	(0.00)
Common border	Binary	0.97	(0.00)	0.28	(0.00)	0.36	(0.00)
Common language	Binary	0.41	(0.00)	0.61	(0.00)	0.57	(0.00)
Common colonizer	Binary	− 1.79	(0.00)	− 1.87	(0.00)	− 3.38	(0.00)
Trade agreements	Binary	0.25 (NS)	(0.76)	− 0.35	(0.00)	− 0.06	(0.00)
Intercept	–	− 16.21	(0.00)	− 18.48	(0.00)	− 21.35	(0.00)
R2		0.59		0.79		0.83	

	Unit and format	Liquefied gas carriers*		Oil tankers*		Ro − ro ships	
GDP_i_	US$ (thousands) - log	1.02	(0.00)	0.94	(0.00)	1.59	(0.00)
GDP_j_	US$ (thousands) - log	1.22	(0.01)	1.27	(0.00)	1.38	(0.00)
Population_i_	log	− 0.25	(0.05)	− 0.55	(0.00)	− 0.83	(0.00)
Population_j_	log	− 0.42	(0.00)	− 0.57 (NS)	(0.22)	− 0.56	(0.00)
Distance	km – log	− 0.73	(0.00)	− 0.72	(0.00)	− 0.37	(0.00)
Common border	Binary	0.41	(0.00)	0.66	(0.00)	0.94	(0.00)
Common language	Binary	0.45 (NS)	(0.33)	0.45	(0.00)	0.35 (NS)	(0.53)
Common colonizer	Binary	− 3.0 (NS)	(0.22)	− 2.15	(0.00)	− 4.17	(0.00)
Trade agreements	Binary	0.12 (NS)	(0.75)	− 0.69	(0.00)	0.59	(0.00)
Intercept	–	− 18.48	(0.00)	− 14.12	(0.00)	− 31.5	(0.00)
R2		0.71		0.68		0.73	

NS indicates the parameters that are not statistically significant for a confidence level of 95%

Energy sectors that will be adjusted to IAMs projections are assigned with a star (*).

Empirical studies have found the GDP coefficient varying between 0.7 and 1.1^33^, which agrees with our findings. Additionally, we perform a hindcast analysis to qualitatively assess the regression based on historical data (SI.3).

Finally, for energy-related trades, we apply an approach that aims at adjusting the shipping demand based on projections of future demand of coal, oil, and gas that have been adopted in previous studies^11^. This is motivated by the uncertainty in how fossil fuel trade will change mid and end-century, from which the SSP-aligned scenarios trends diverge from historical trends, in particular, because around 50% of global oil supply, 20% of coal’s and 10% of natural is supplied by seaborne trade^11^. Through this approach, we expect to capture the dynamics of the impact of future energy trade on shipped freight. For that, we use the expected demand for fossil fuels in SSP-RCP scenarios from IAMs to adjust global energy demand while maintaining the same trade patterns between countries—assuming that supply will still exist as reservoirs are phased out. For more details on the implementation, see previous works with the MariTeam model^38^.

**Figure 1:**
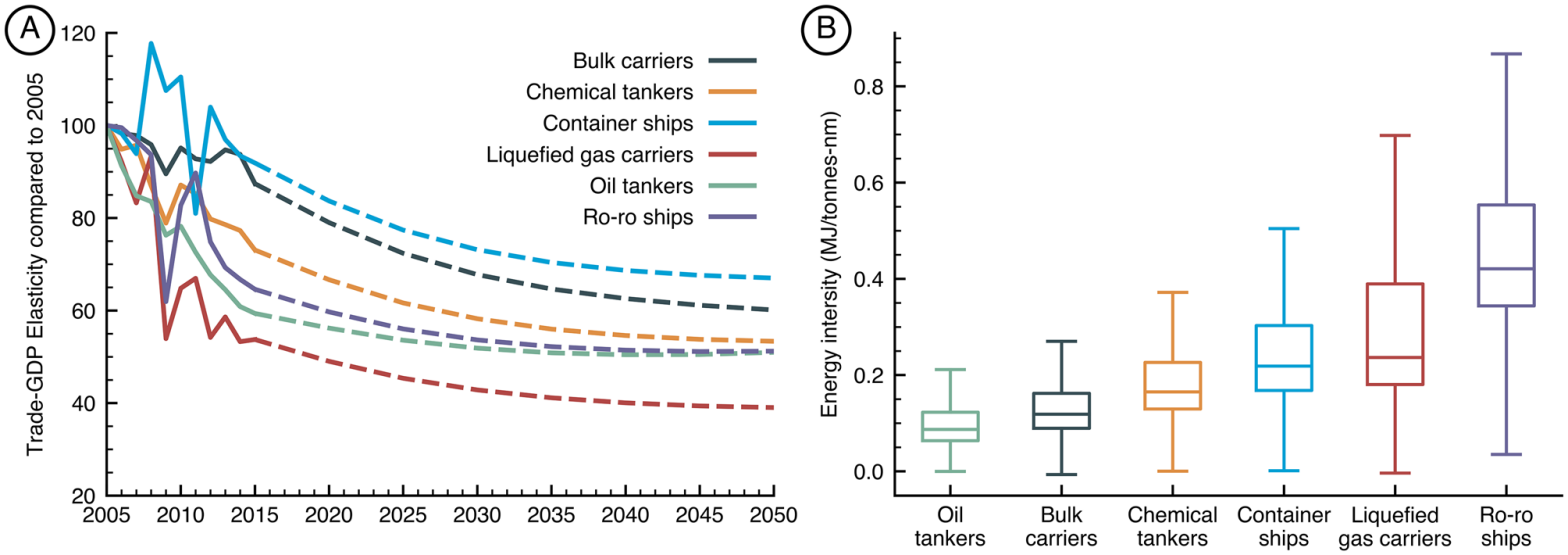
Trade projections at regional and ship type levels and energy demand implications. Figure 1A shows the hindcast analysis along with projects for the 11 MESSAGE regions (dashed line), in combination with historical data (solid line). Figure 1B shows the change in Trade-GDP ratio for global trade when aggregated in different ship types.

Additionally, we go beyond the classic formulation of the gravity models by including the changes in trade-GDP elasticity that are on decline especially after the 2010s. Figure 1A shows in the solid lines the historical elasticity for all ship types. In that, one can see the 2007–2008 economic crisis’s impact on trade as well the recent decline in the elasticity. Performing this analysis at a ship type level, we find, for example, that container ships and bulk carriers have a lower elasticity (change in carrier trade volume / change in global GDP), whereas others like oil and gas carriers have suffered a more drastic change. Based on this historical data, we develop projections until 2015 using logarithm regressions to represent a decay that accelerates rapidly at first and then slows over time. For more information on the regression, see SI.1. The trade-GDP elasticities (TGE) obtained for each given year are used to adjust the bilateral trade projections (F_ij_) obtained originally from the gravity model as shown in Eq. (4).

4 $${{{\text{F}}}_{{\text{ij}}}}_{{\text{year}}}={{{\text{F}}}_{{\text{ij}}}}_{2015}\cdot {{\text{TGE}}}_{{\text{year}}}$$

### Shipping spatial projections

The spatial dimension of shipping is included through the full bottom-up MariTeam framework^34,37^ (Maritime Transport Environmental Assessment Model), which combines ship technical specifications, ship location data, and weather data in high spatial and temporal resolution obtained from satellite data (AIS) for approximately 50,000 vessels. The model performs ship resistance calculations for each data point providing energy demand estimations along routes that vary according to the speed and the weather conditions. Through extensive big-data processing, we transform this collection of points into meaningful port-to-port routes that allow us to estimate energy demand for six shipping segments: bulk carriers, chemical tankers, container ships, general cargo ships, liquefied gas tankers, oil tankers, and ro-ro ships. This approach allows us to account for the occupancy of each segment (laden versus ballast voyage ratios) and the energy intensity across different regions that often stems from adverse weather conditions, shorter routes that require ships to stay longer at the port, and routes composed of smaller vessels that commonly have a higher energy intensity. Aggregated data for ship types is shown in Fig. 1B, demonstrating the importance of taking into account differences in energy intensity across the fleet. Additionally, the heterogeneity of energy intensity is illustrated in SI.2 for the top 50 O&D in energy demand. For each O&D, thus, we have a trade network containing the following levels of information for each of the six ship segments:

- Historical trade from 1995 to 2018 based on BACI trade flows.
- Energy demand for primary and auxiliary engines based on AIS data.
- Transport work, which is the amount of cargo transported over distance sailed, considering occupancy percentages.

## Results

### Trade projections

**Figure 2:**
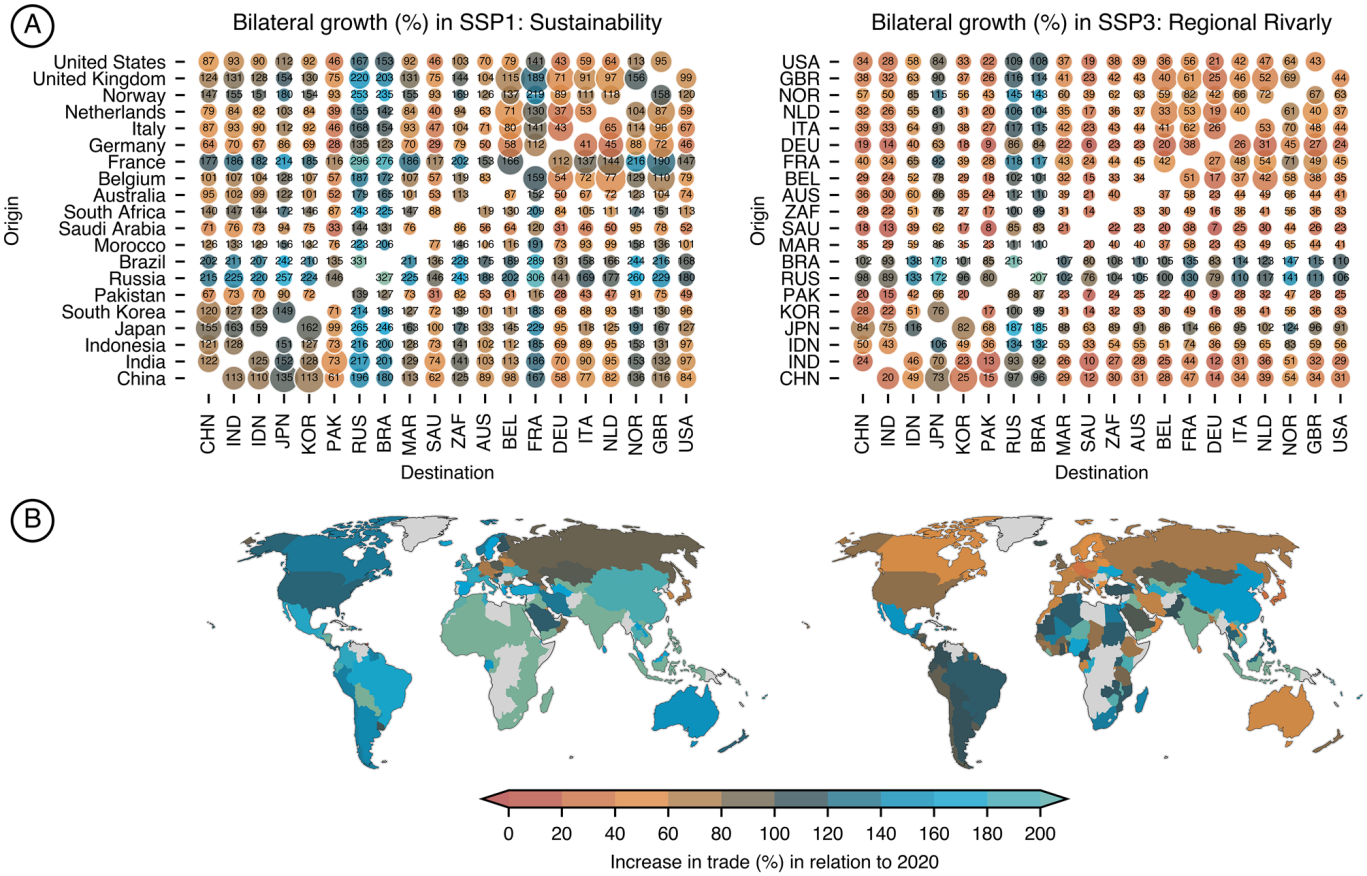
Trade growth projections for SSP1-1.9 and SSP3-7.0 GDP and population projections for 2050. Upper Fig. 2A show bilateral growth for pairs of origin and destination countries — values and colors indicate the increase in trade by 2050 whereas, the circle radius indicates the stylized size of bilateral trade in 2050. Figures 2B represent the individual country’s growth of maritime trade by 2050 under the same SSP1-1.9 and SSP3-7.0 configurations.

Applying the results from the gravity model, we can calculate the bilateral growth projections of trade between 138 countries (Fig. 2). In Fig. 2A, one can identify the different patterns in trade between an SSP1-1.9 and SSP3-7.0 scenario, especially the lower growth rate of developed countries, e.g., Europe and North America, and the rise of developing countries such as BRICS and Asia due to the high GDP and population growth. That is reinforced in Fig. 2B, which shows the difference in single-country trade growth between the global north and global south, especially for an SSP3-7.0 scenario.

### Energy demand projections

**Figure 3:**
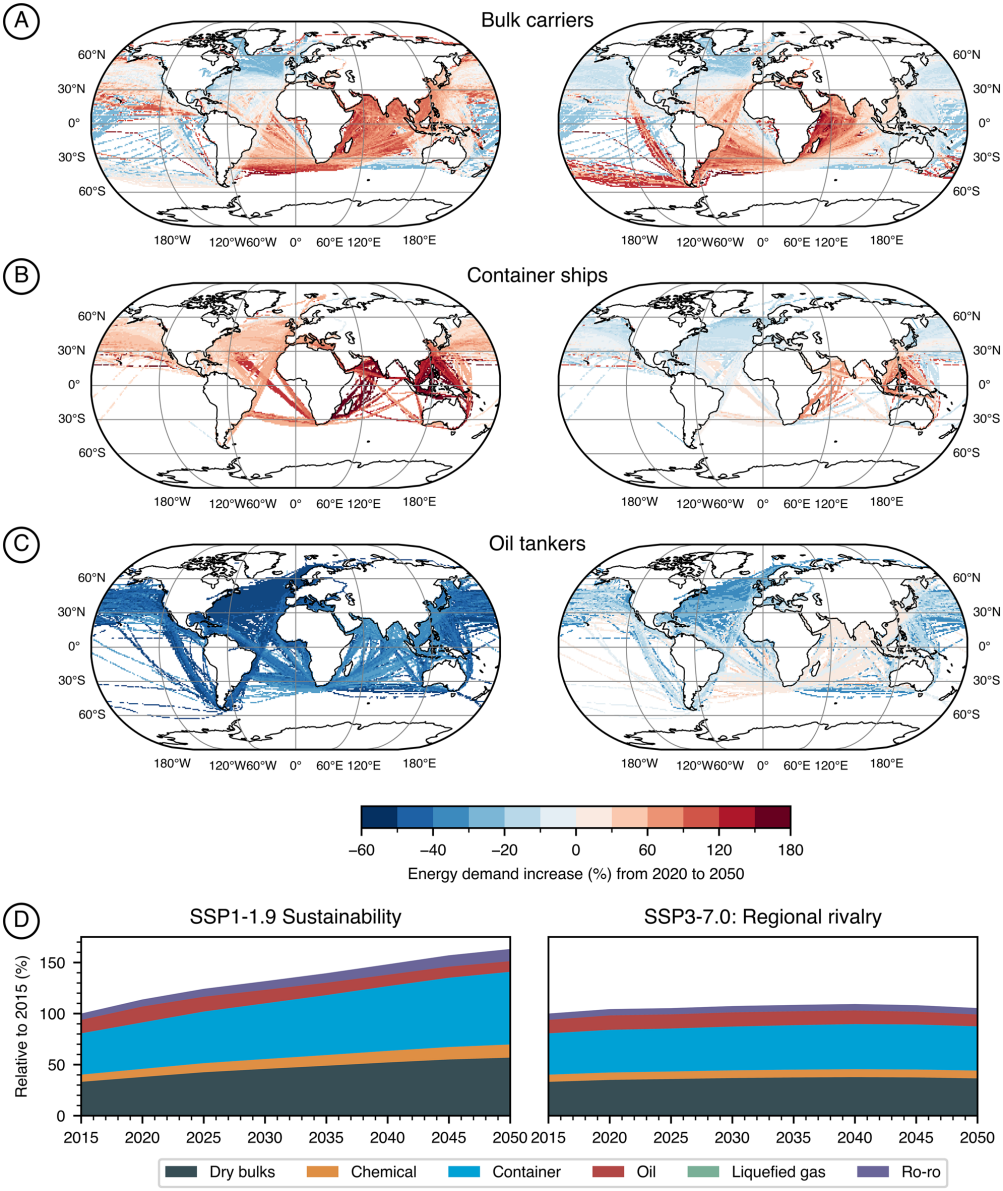
Relative growth of international shipping between years 2050 and 2015. Panel A displays the transport-work projections relative to the year 2015 for SSP1-1.9 and SSP3-7.0 projections where B shows the respective change in energy demand for the sector in the same sector that is highly influenced by the energy intensity of each ship type. Figure C shows the spatially explicit increase in energy demand with higher rates in the southern hemisphere driven by the higher economic growth of Asian countries.

**Figure 4:**
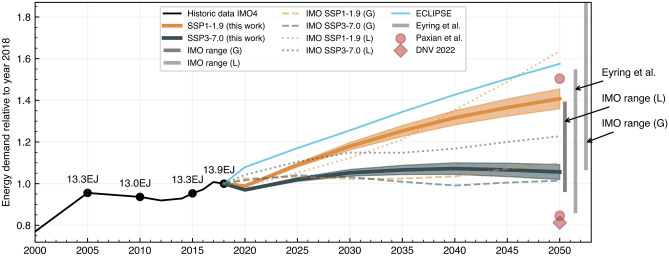
Total energy demand projections for shipping between 2018 and 2050. Results are presented for SSP1-1.9 and SSP3-7.0, when available scenarios compared with similar studies given as trajectories, single-year estimations, or ranges. IMO results are presented for the gravity model (G) approach and the logarithm method (L)^5^. All values are indexed to match the historical value of 13.9 EJ in 2018 — historical data based on the 4th IMO GHG study.

Combining the trade growth of each O&D given by the results from the gravity model with the energy demand for the same O&D in the MariTeam model, we can obtain the global energy demand for shipping until 2050. See Fig. 4 for a comparison with similar studies with numbers normalized to the year 2018. In addition, based on the standard deviation in Table 1, we can also provide high and low estimates established in the gravity model regression in order to address uncertainty in the projections (shaded area in Fig. 4). Results indicate that an SSP1-1.9 scenario could increase energy demand by 44%, whereas an SSP3-7.0 scenario would increase by 3%. In general, results agree with the current literature, even though the mid-term trend towards 2050 differs in some cases (stabilization versus continuous growth). Moreover, we show a different pattern in the projections compared to the IMO. In the SSP1-1.9 scenario, our projections indicate that shipping could reach a phase of saturation towards 2050 as compared to steady growth from the IMO projections. For the SSP3-7.0, we identify a trend of energy demand reduction. By utilizing narratives aligned with the SSP framework, these results endogenize the wide range of possible maritime energy demand projections strictly following an SSP1-1.9 or SSP3-7.0 narrative. With this bottom-up set of energy demand levels, we go beyond existing SSP-specific projections, such as the ones introduced by the IMO (Fig. 3), to fully internalize the underlying SSP narrative and ensure a consistent interpretation and linkage to IAMs and ESMs. That is possible through the trade-GDP elasticities incorporated into the gravity model and the use of detailed ship energy demand instead of transport-work metrics.

## Discussion

To overcome the challenges in mitigating climate change, significant efforts will need to be pursued in the shipping industry. The attainability of emission reduction goals depends, however, on the demand the sector will experience due to global economic growth and change in trade patterns. To tackle the uncertainty in that, the SSP-RCP framework allows us to explore a wide range of possible futures that lead to significantly diverse challenges towards decarbonizing shipping. According to our projections, shipping could increase its overall energy demand by 44% by 2050 in an SSP1-1.9 scenario, compared to 3% in SSP3-7.0 scenario.

Despite the lower energy demand of an SSP3-7.0 scenario, one must consider the global implications of a scenario dominated by regional rivalry that could affect the collaboration between actors in reducing the dependency on fossil fuels and increasing the energy efficiency in the sector. Likewise, a sustainability-driven SSP1-1.9 scenario, even though requiring more energy, might enable new solutions to provide clean energy for shipping and higher efforts to increase the energy efficiency in the sector. Ship segments follow different pathways under each scenario, for example, the reduction in transport-work from energy carriers obtained in SSP1-1.9 could be nearly offset by the increase in demand in other sectors like bulk carriers and container ships compared to the SSP3-7.0 projections (Fig. 3). Thus, we highlight the importance of actions and policies targeting the whole sector as well as specific segments.

However, in these scenarios, if technical efficiency goals established by the industry are not met, e.g., reducing carbon intensity by 40% by 2030 compared to a 2008 baseline, the energy demand could increase substantially beyond the estimates presented here. Alternatively, if technical and operational efficiency improvements^35^ are pursued across the fleet, the sector could achieve energy demand lower than presented in our scenarios, and reducing to even lower than the current energy demand in the shipping sector.

It is important to note that the scenarios presented in this study contain limitations and uncertainties that should be addressed in further studies. Amongst them, economic growth, trade patterns and globalization trends are highly uncertainty, especially considering geopolitical factors and regional conflicts that have increased in number and scale in the recent years. Developments in ship energy efficiency (which have not been addressed in this study) along with adoption rate of new technologies across the fleet can also impact the energy demand projections. On the supply side, a world fueled by renewable energy with decentralized production of fuels rather than centralized extraction of fossil fuels could impact the demand of energy carriers in shipping, which cannot be fully assessed in this study. Lastly, one should consider the adoption of the updated SSP Socioeconomic Projections released in 2024 in future works.

**Figure 5:**
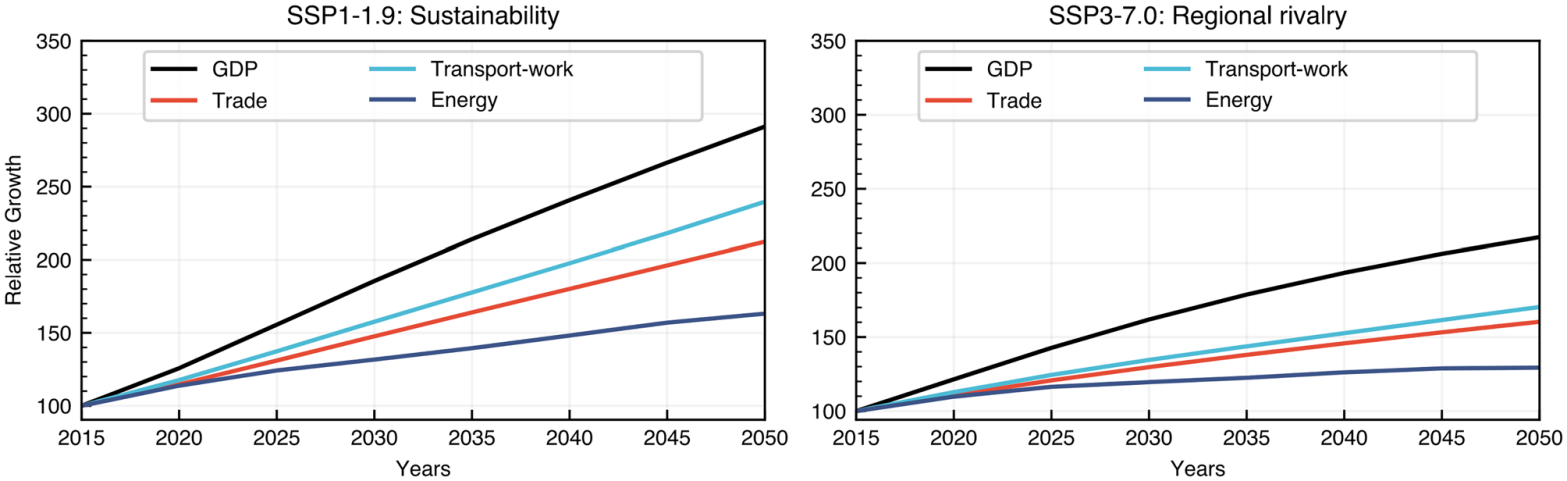
Difference in growth projections from a 2015 baseline.

When we compare our results with the literature, we find that our projections generally align with previous results (Fig. 4), confirming the hypothesis that north–south trade will grow faster than existing main trades, especially east–west (Fig. 3), reaching a level of saturation in the long-term^12^. The major difference being the demand around the 2050s (Fig. 4), mainly because previous studies have assumed trade could continue to grow at the same pace as today, which is a phenomenon gradually changing as the elasticity of GDP-trade decreases (see Fig. 1b). This could impact the shipping inventories currently used in climate modeling, especially when considering the substantially higher ship traffic toward Asian countries when comparing SSP1-1.9 and SSP3-7.0 scenarios (Fig. 3). Thus, our projections provide a revised perspective on energy demand to the shipping sector, often much lower than previous studies, making goals to decarbonize the sector more attainable if compared with previous studies. On the other hand, our more conservative scenarios show that reducing energy demand by increasing energy efficiency is paramount to have enough renewable fuels available shipping. For example, in a world that will have 8–35 EJ^36^ of green hydrogen available by 2040, a scenario in which shipping alone will consume 20 EJ could jeopardize decarbonization goals. Thus, we believe that including fine spatial resolution allows us to model the increase in emissions across different ship types and regions of the globe. Moreover, with this study, we make those inventories available to the scientific community to fill the need for detailed, high-resolution, and transparent models that are essential to improve the integration with other research fields in climate sciences (Figs. 4 and 5).

### Supplementary Information

Supplementary Information.

## Data Availability

The gravity model and the data used in this study are not open source but can be provided upon reasonable request for the corresponding author of this publication at diogo.kramel@ntnu.no.
