# Supplementary material for: Advancing SSP-aligned scenarios of shipping toward 2050
Source: Sci Rep. 2024 Apr 18;14:8965. doi: 10.1038/s41598-024-58970-3 (PMC11026375; doi:10.1038/s41598-024-58970-3)

Supporting Information of:

Advancing SSP-aligned Scenarios of

Shipping Toward 2050

Diogo Kramel*^1^, Sebastian Marco Franz^2^, Jan Klenner^1^, Helene Muri^1^, Marie Munster^2^, and Anders H. Stromman^1^

^1^Industrial Ecology Programme (IndEcol), Norwegian University of Science and Technology (NTNU), Trondheim, Norway

^2^Department of Technology, Management and Economics, Technical University of Denmark (DTU), Copenhagen, Denmark

* Corresponding author: diogo.kramel@ntnu.no

**SI.1 – Trade elasticity**

Trade-GDP elasticity logarithmic regressions for ship types and their respective R2.

$$y=a\log\left( \mathrm{year} \right)+b$$

| **Ship type** | **a** | **b** | **R2** |
| --- | --- | --- | --- |
| Bulk carriers | -3.93 | 100.58 | 0.61 |
| Chemical tankers | -10.72 | 102.29 | 0.85 |
| Container ships | -2.71 | 103.79 | 0.39 |
| Oil tankers | -16.89 | 103.17 | 0.95 |
| Liquefied gas carriers | -21.80 | 105.09 | 0.79 |
| Ro-ro | -15.78 | 106.91 | 0.63 |

**SI.2 – Energy intensity and demand for highest 50 O&Ds**


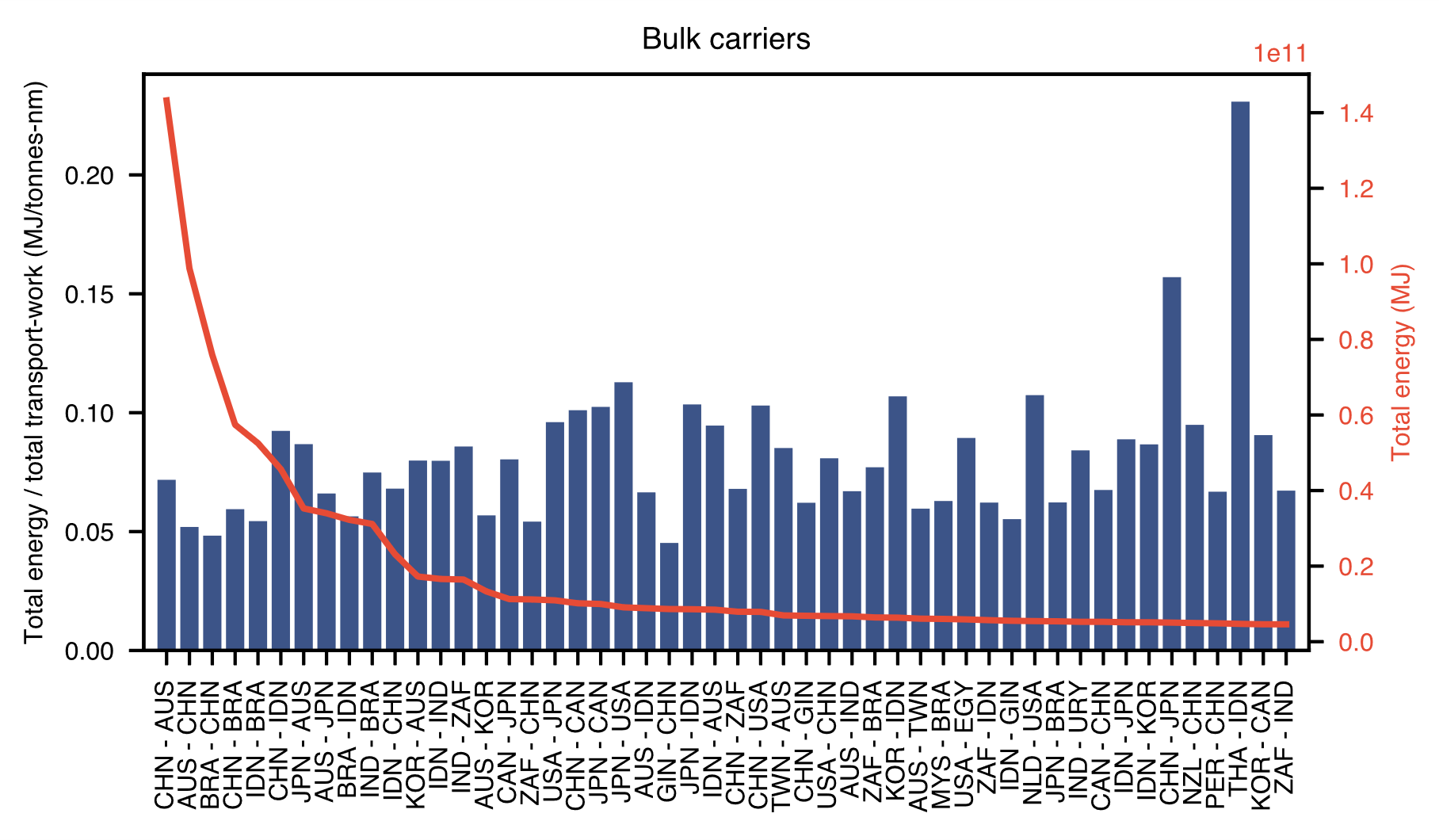
**
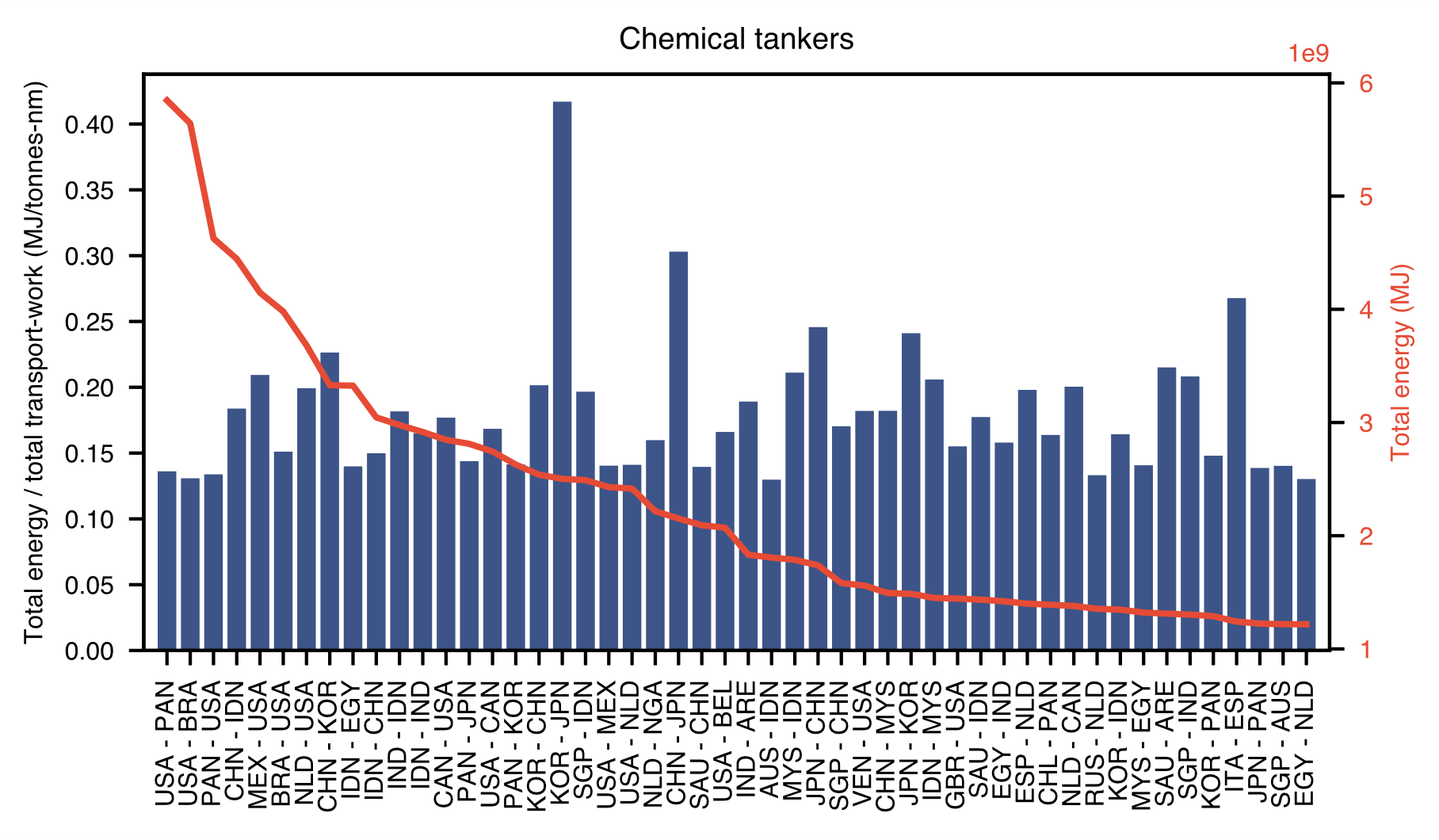
**

**
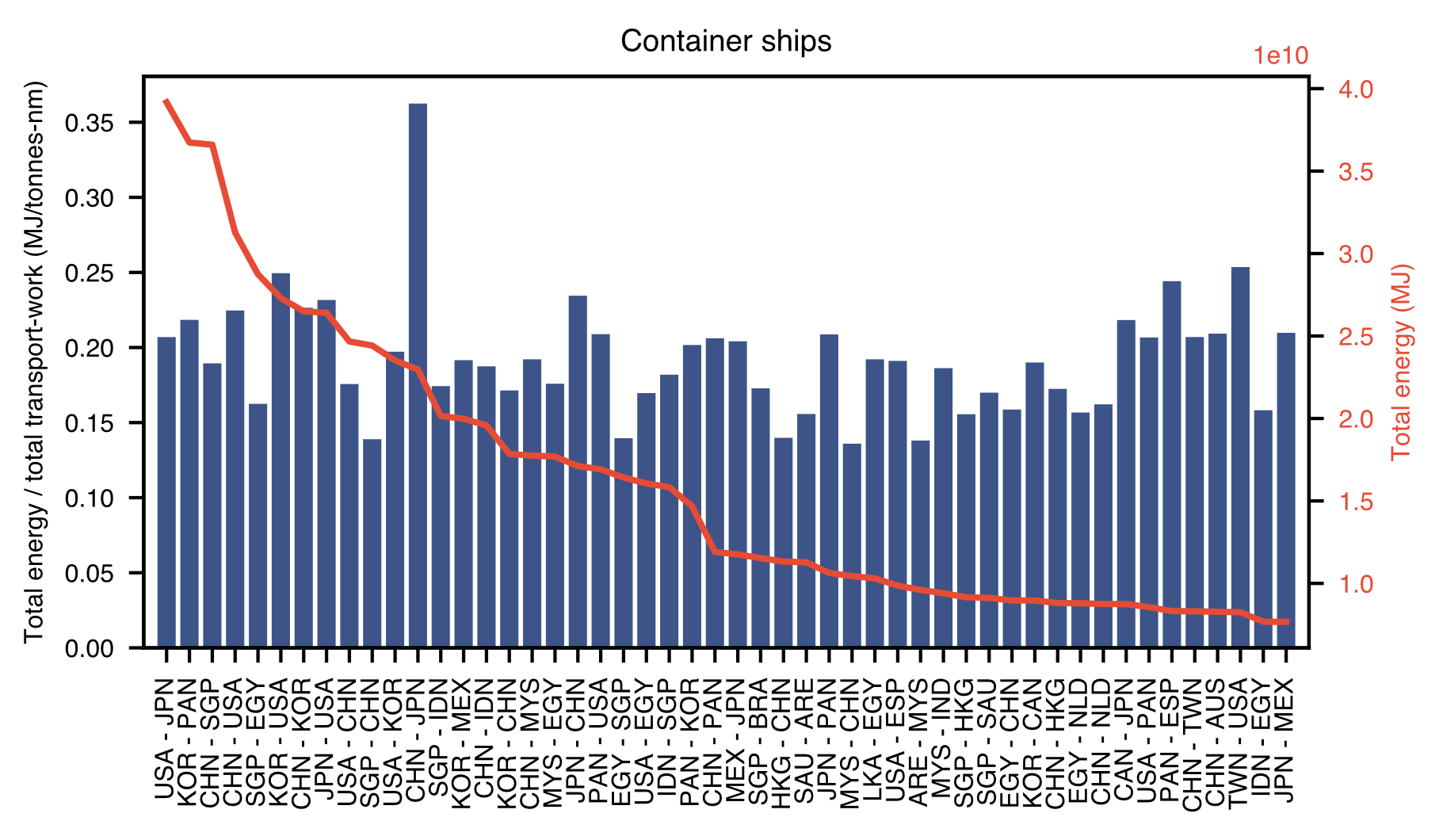
**
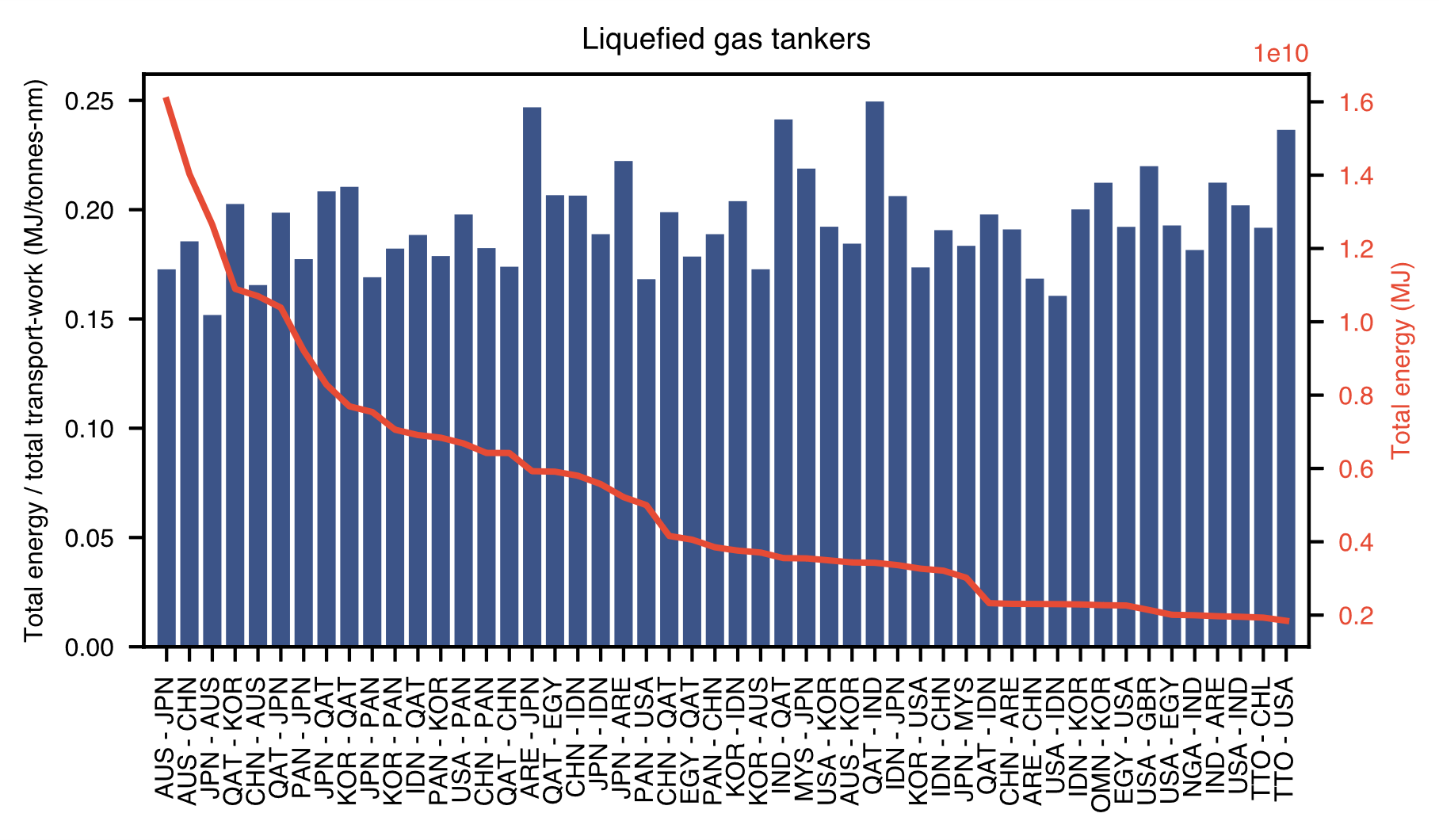

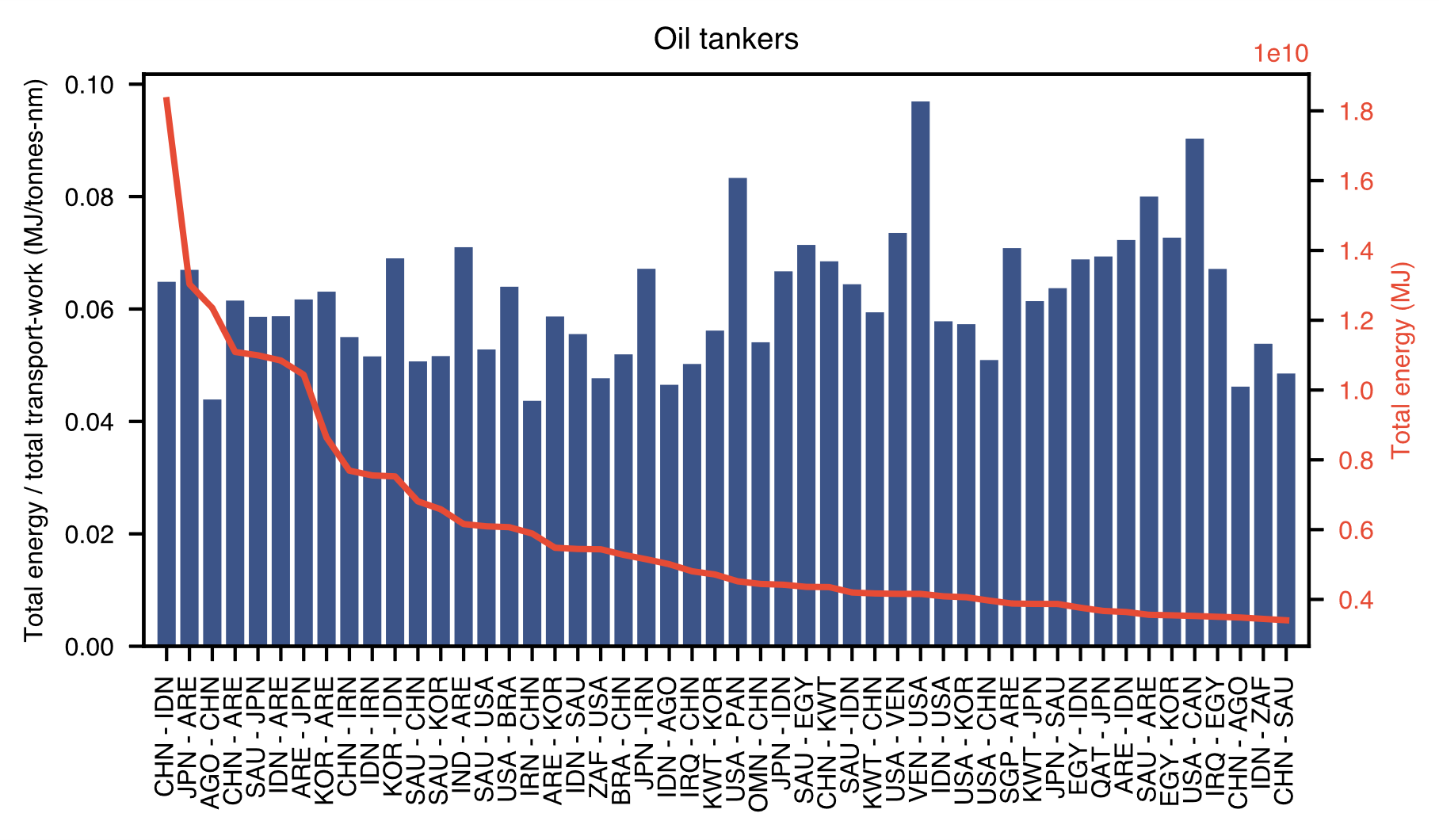

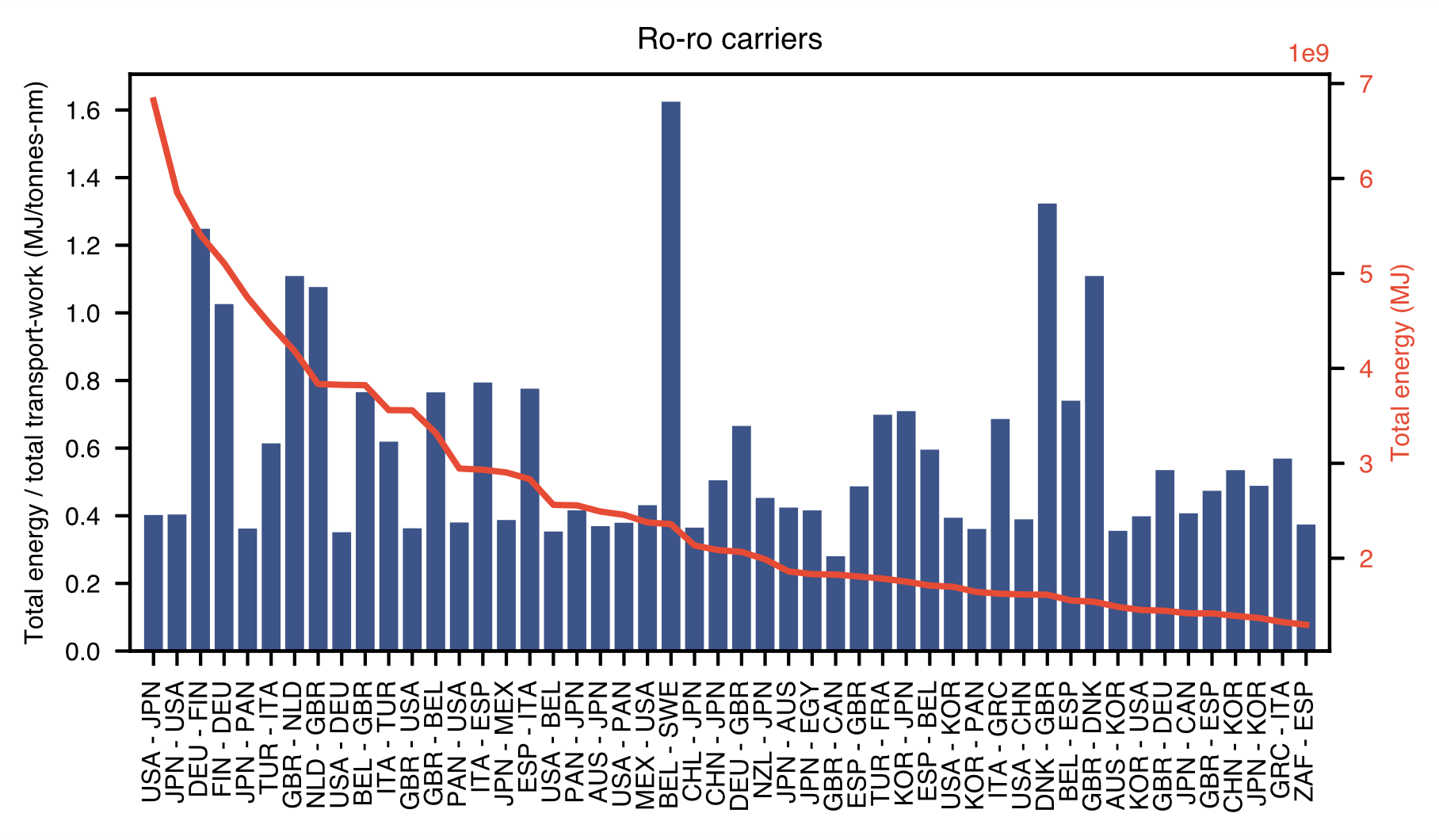


**SI.3 – Hindcast analysis and projections for R11 regions**


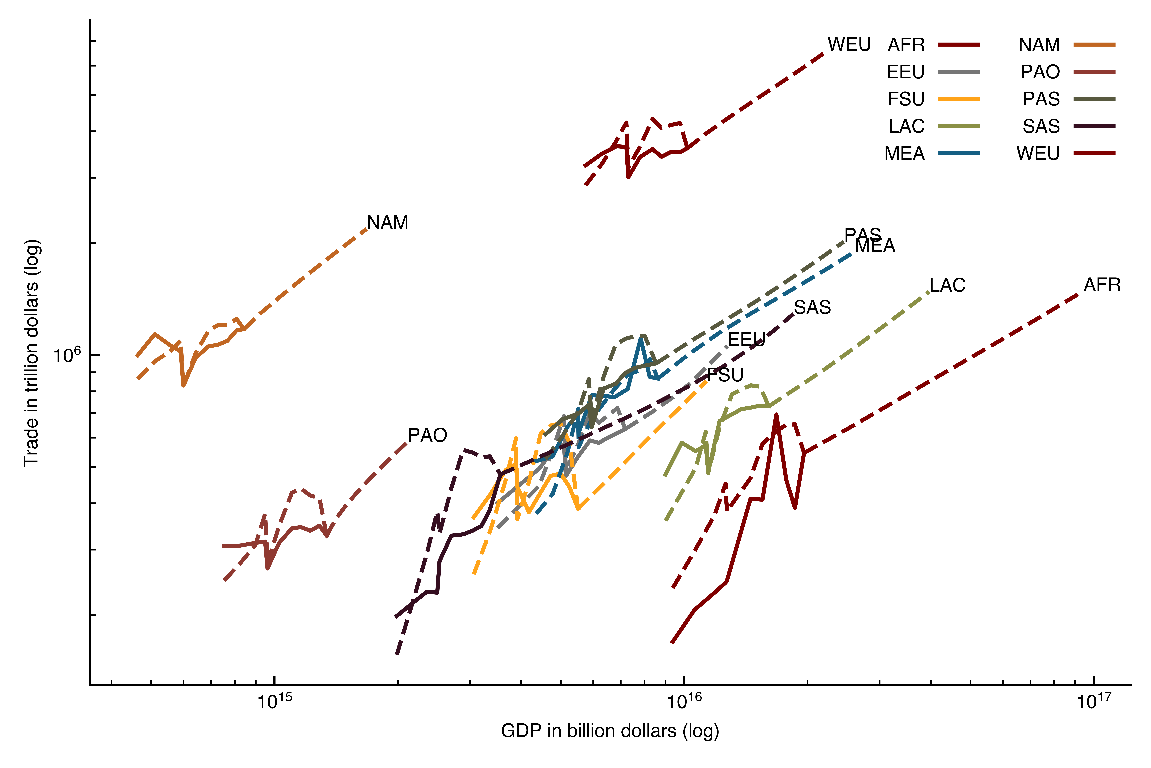

Supplement: Supplementary file 1 — Supplementary Information. [file 41598_2024_58970_MOESM1_ESM.docx]
